# Elevated C‐Reactive Protein–Albumin–Lymphocyte Index Is Associated With Reduced Mortality in Chronic Inflammatory Airway Diseases: A Population‐Based Observational Study

**DOI:** 10.1155/mi/8257027

**Published:** 2026-08-26

**Authors:** Yuting Fan, Zhao Chen, Mingshan Xue, Wenqiang Li, Youli Wen, Baoqing Sun, Zhiping Deng

**Affiliations:** ^1^ Department of Clinical Medicine, North Sichuan Medical College, Nanchong 637000, Sichuan, China, nsmc.edu.cn; ^2^ Department of Respiratory and Critical Care Medicine, Zigong First People’s Hospital, Zigong 643000, Sichuan, China; ^3^ Department of Clinical Laboratory, The First Affiliated Hospital of Guangzhou Medical University, Guangzhou 510000, Guangdong, China, gzhmc.edu.cn

**Keywords:** CALLY index, CIAD, mortality, NHANES, population-based study

## Abstract

**Background:**

The C‐reactive protein–albumin–lymphocyte (CALLY) index, a novel composite marker derived from lymphocyte count, serum albumin, and C‐reactive protein (CRP) levels, is commonly used to assess nutritional and inflammatory status. However, existing studies have not clarified the association between the CALLY index and prognosis in patients with chronic inflammatory airway diseases (CIADs). This study aims to elucidate the relationship between the CALLY index and the risks of all‐cause and cardiovascular mortality among CIAD patients in the United States.

**Methods:**

This investigation included 4128 patients with CIAD from the National Health and Nutrition Examination Survey (NHANES, 1999–2010). The CALLY index was measured only at baseline. We employed weighted Cox proportional hazards regression models to assess the association between the CALLY index and risks of all‐cause and cardiovascular mortality across three progressively adjusted models. To evaluate potential nonlinear dose–response relationships, we applied restricted cubic spline (RCS) models. Additionally, subgroup analyses and sensitivity analyses were conducted to verify the robustness of our findings.

**Results:**

Multivariable‐adjusted Model 3 demonstrated that, compared with the lowest quartile, the highest CALLY index quartile showed a significant inverse association with both all‐cause and cardiovascular mortality, with corresponding HRs of 0.36 (95% CI: 0.27–0.47) and 0.33 (95% CI: 0.21–0.51), respectively (both *p*  < 0.05).

**Conclusions:**

The CALLY index showed a significant inverse association with all‐cause and cardiovascular mortality among U.S. adults with CIAD, suggesting its potential value as a prognostic tool for risk stratification in this group.

## 1. Background

Asthma, chronic bronchitis, and chronic obstructive pulmonary disease (COPD) are collectively categorized as chronic inflammatory airway diseases (CIADs) [1]. These disorders contribute to an increasing global health burden and pose substantial challenges to public health systems [2, 3]. In 2017, an estimated 3.91 million global deaths were attributable to chronic respiratory diseases, ranking as the third leading cause of mortality (7.0% of total deaths), surpassed only by cardiovascular diseases and cancer, marking an 18% rise compared with the figures recorded in 1990 [3]. As the most common chronic respiratory disorders, COPD and asthma are strongly associated with persistent airway inflammation. The 2019 Global Burden of Disease According to the report, asthma is a complex clinical illness that affects over 300 million people worldwide and accounts for 455,000 fatalities. COPD was expected to impact 212.3 million people, resulting in 3.3 million deaths [4, 5]. These staggering statistics underscore the imperative for early detection and timely intervention to mitigate the mortality risks associated with CIAD.

Continuous inflammation in the airways is usually triggered by the combined effect of genetic susceptibility and environmental factors, and it is the core of the pathogenesis of CIADs [6]. The main features of the inflammatory response include an increase in the number of recruited alveolar macrophages, neutrophils, T lymphocytes (mainly TC1, TH1, and TH17 cells), and innate lymphoid cells in the circulation. These immune cells interact with structural cells, such as epithelial cells, endothelial cells, and fibroblasts, and emit numerous proinflammatory mediators, such as cytokines, chemokines, growth factors, and lipid mediators, which impact airway function [7]. Research has confirmed that the upregulation of inflammatory markers, such as C‐reactive protein (CRP), neutrophil, and lymphocyte counts, is closely related to increased susceptibility to CIAD [8]. Thomsen et al. [9] found that in patients with COPD, if CRP, fibrinogen, and white blood cell count are elevated, their risk of acute exacerbation also increases; this trend persists even in patients whose condition is not severe and who have no history of acute exacerbations. The neutrophil‑to‑lymphocyte ratio (NLR) and neutrophil‑to‑albumin ratio (NPAR) are easily obtainable inflammatory composite parameters. A large‐sample investigation by Lan et al. [10] reveals that these two indicators not only predict the risk of COPD but also demonstrate good predictive performance in the early identification of acute exacerbations, thereby holding significant clinical value. However, existing studies only indicate that these inflammatory and immune markers mainly provide single or partial combined diagnostic and prognostic information, while the relationship between composite markers and CIAD has not been fully explored [11].

The C‐reactive protein–albumin–lymphocyte (CALLY) index, calculated by combining three parameters—lymphocyte count, serum albumin, and CRP—is a novel composite biological marker that simultaneously reflects both the body’s nutritional status and inflammation levels and holds significant clinical value for assessment. Previous studies have identified significant links between the CALLY index and multiple clinical conditions, such as sarcopenia and cardiorenal syndrome [12, 13]. Additionally, the CALLY index plays a crucial role in evaluating the survival prognosis and treatment response of various tumor patients [14]. Despite these research findings, the association between the CALLY index and CIAD remains largely unexplored. Clarifying this association will offer practical guidance for clinical decision‐making. In response to this research gap, this study utilized population‐based data from the 1999–2010 National Health and Nutrition Examination Survey (NHANES) in the United States to analyze the association between the CALLY index and mortality events related to asthma, chronic bronchitis, and COPD, with the aim of providing reliable epidemiological evidence to inform prevention strategies and long‐term management plans for chronic airway inflammatory diseases.

## 2. Methods

### 2.1. Data and Study Participants

**Figure 1:**
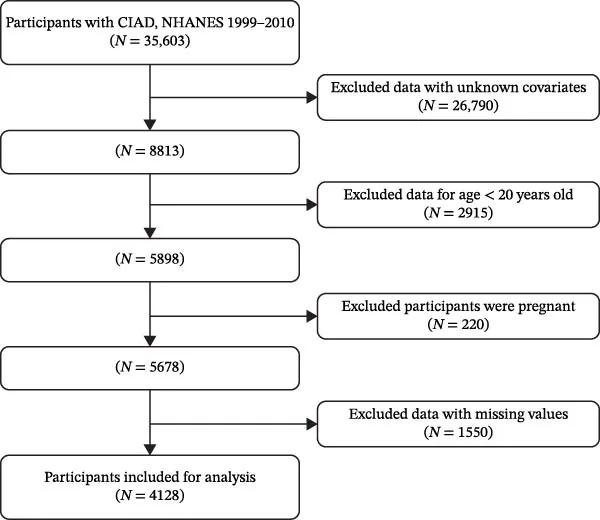
Study flow diagram illustrating the participant inclusion and exclusion process.

Large‐scale data from the NHANES database were used in this study to assess the nutritional status of the noninstitutionalized U.S. population. The data were collected via laboratory tests and physical tests in transportable examination facilities, in addition to structured interviews conducted in the participants’ homes. The survey was performed in two‐year cycles and employed a multistage random sampling approach to ensure that the sample accurately represented the U.S. population. All participants provided written informed consent, in compliance with the guidelines of the National Center for Health Statistics Research Ethics Review Board (NCHS ERB). This dataset is publicly available at https://www.cdc.gov/nchs/nhanes/. For the present analysis, we extracted NHANES data from the 1999 to 2010 survey cycles. Eligible participants were adults aged ≥20 years with a diagnosis of CIAD and complete data on CRP, albumin, and lymphocytes for the CALLY index calculation. The study initially enrolled 35,603 CIAD patients. After eliminating pregnant individuals, those with missing variables, and those with partial data on critical outcomes, 4128 patients satisfied the inclusion requirements and participated in the final analysis. The screening process is depicted in Figure 1.

### 2.2. Calculation of the CALLY Index

The CALLY index was calculated as albumin (g/L) × lymphocytes (10^9^/L) ÷ [CRP (mg/L) × 10], and full blood counts were obtained via the Coulter DxH 800 analyzerundern the guidance of qualified staff [12]. Patients were divided into four groups according to CALLY index quartiles, where lower quartiles reflected a greater inflammatory burden (Q1: 1.71–141.02) and higher quartiles indicated milder inflammation (Q4: 860.25–170640).

### 2.3. Study Definition of CIAD

For the present investigation, CIAD encompassed participant‐reported asthma, chronic bronchitis, and COPD. Study participants responded to queries asking whether physicians or other medical professionals had diagnosed them with active or prior asthma, chronic bronchitis, or COPD. Individuals providing affirmative responses to any question were categorized as having relevant airway disorders [15].

### 2.4. Assessment of Mortality Risk

Using the National Death Index (NDI; https://www.cdc.gov/), we retrieved mortality information with follow‐up through December 31, 2019. Records for overall and cardiovascular mortality were extracted from this database. Fatal cardiovascular events were ascertained according to the International Classification of Diseases, 10th Revision (ICD‐10) codes I00–I09, I11, I13, and I20–I51 [16]. The final analytical cohort contained 1068 overall deaths and 362 cardiovascular fatalities.

### 2.5. Covariates

The study incorporated potential confounding covariates selected from established literature, including age (categorized as 20–44, 45−64, and ≥ 65 years); sex (male/female); race/ethnicity (white/others); marital status (married/partnered, never married, or divorced/widowed/separated); educational attainment (less than high school, high school, or beyond high school); poverty–income ratio (PIR, calculated per U.S. Department of Health and Human Services guidelines by dividing family income by poverty thresholds specific to household size and categorized as ≤1.0, 1.1–3.0, and >3.0) [17]; insurance status (insured/uninsured); smoking status (never, former, or current smoker) [18]; alcohol consumption (nondrinker/drinker); body mass index (BMI, <25.0, 25.0–30, and ≥30 kg/m^2^); diabetes status (borderline, diabetic, or nondiabetic); hypertension (yes/no); cardiovascular disease (yes/no); healthy eating index‐2015 (HEI‐2015) scores and daily energy intake (both analyzed by quartiles).

### 2.6. Statistical Methods

In accordance with the NHANES analytical guidelines, we applied sampling weights to all statistical analyses. Continuous indicators were transformed into categorical forms and summarized as counts (percentages). We categorized CALLY scores into four levels based on quartiles using the first quartile (Q1) as the reference point. Then, to assess the relationship between CALLY quartiles and CIAD mortality, we constructed a series of multivariate Cox proportional hazard models and calculated the corresponding hazard ratios (HRs) and their 95% confidence intervals (CIs). Three models were constructed with adjustments for different covariates to provide a more comprehensive analysis, and we also used a restricted cubic spline (RCS) model to better evaluate the dose–response pattern, setting the knots at the 10th, 50th, and 90th CALLY percentiles to assess the nonlinear relationship with mortality risk. Additionally, stratified analyses were conducted across various subgroups to examine the effect of CALLY on all‑cause and cardiovascular death risk among CIAD patients in various population subgroups. We excluded cases with follow‐up durations ≤ 2 years to control for survival‐time bias and removed cancer‐related deaths to minimize competing risk interference so as to ensure the reliability of the results. Further sensitivity analyses were conducted to confirm the stability of our findings. For CIAD patients, multivariate Cox regression risk models were constructed using CALLY score quartiles (adjusted for covariates such as age, sex, and comorbidities) to evaluate their independent predictive effect on all‐cause mortality and cardiovascular‐specific mortality.

We ran all statistical analyses using R (Version 4.3.3) and set statistical significance at a two‑tailed *p*  < 0.05.

## 3. Results

### 3.1. Participant Baseline Characteristics

After screening, a total of 4128 participants (43.46% male) from the NHANES 1999–2010 database satisfied the inclusion standards. Table 1 presents the baseline characteristics of the study population by CALLY quartiles. Most participants excluded due to ineligibility were under 20 years of age, primarily due to a lack of relevant health survey data or a failure to meet eligibility criteria. The majority of the participants were white (2493, 77.91%), married or partnered (2373, 60.41%), and had attained at least a high school education (2015, 55.92%), with a substantial proportion reporting being never‐smokers (1697, 42.82%). Comparative analysis across CALLY index quartiles exhibited significant differences (*p* < 0.05) across all baseline characteristics, except for smoking status and insurance coverage.

**Table 1:** Participant baseline characteristics.

Characteristics	Total	Quartiles of CALLY index							*p*‐Value
		Q1 (1.71–141.02)		Q2 (141.02–337.80)		Q3 (337.80–860.25)		Q4 (860.25–170640)	
*N*	4128	1032		1032		1032		1032	—
Age group, *n* (%)	—	—		—		—		—	<0.0001
≤44	1575 (45.23)	302 (36.12)		334 (39.69)		406 (46.38)		533 (55.87)	—
45–64	1418 (36.47)	374 (37.43)		374 (39.11)		358 (36.41)		312 (33.52)	—
≥65	1135 (18.31)	356 (26.45)		324 (21.20)		268 (17.21)		187 (10.61)	—
Gender, *n* (%)	—	—		—		—		—	<0.0001
Female	2243 (56.54)	644 (67.95)		597 (61.09)		529 (52.09)		473 (47.89)	—
Male	1885 (43.46)	388 (32.05)		435 (38.91)		503 (47.91)		559 (52.11)	—
Race, *n* (%)	—	—		—		—		—	0.04
Other	1635 (22.09)	436 (25.51)		413 (23.06)		402 (19.75)		384 (20.71)	—
White	2493 (77.91)	596 (74.49)		619 (76.94)		630 (80.25)		648 (79.29)	—
Marital status, *n* (%)	—	—		—		—		—	<0.0001
Married/Living with partner	2373 (60.41)	554 (56.63)		604 (62.11)		607 (59.63)		608 (62.49)	—
Never married	639 (16.53)	135 (14.33)		113 (11.61)		159 (17.47)		232 (21.60)	—
Widowed/Divorced/Separated	1116 (23.07)	343 (29.04)		315 (26.28)		266 (22.90)		192 (15.92)	—
Educational attainment, *n* (%)	—	—		—		—		—	<0.0001
High school diploma	985 (24.79)	261 (28.65)		243 (24.10)		249 (24.81)		232 (22.42)	—
Lower than high school	1128 (19.29)	327 (23.78)		296 (20.38)		295 (20.09)		210 (14.26)	—
More than high school	2015 (55.92)	444 (47.57)		493 (55.52)		488 (55.10)		590 (63.32)	—
Poverty income ratio group, *n* (%)	—	—		—		—		—	<0.0001
≤1	1332 (24.61)	402 (31.92)		320 (24.26)		318 (22.48)		292 (21.25)	—
1.1–3.0	1530 (34.18)	383 (37.94)		383 (33.54)		383 (35.05)		381 (31.12)	—
＞3	1266 (41.21)	247 (30.14)		329 (42.20)		331 (42.47)		359 (47.63)	—
Insurance, *n* (%)	—	—		—		—		—	0.31
No	715 (16.66)	156 (15.38)		158(16.83)		184(15.13)		217(18.81)	—
Yes	3413 (83.34)	876 (84.62)		874(83.17)		848(84.87)		815(81.19)	—
Smoking, *n* (%)	—	—		—		—		—	0.15
Former	1273 (28.55)	354 (33.26)		331 (28.67)		313 (28.19)		275 (25.20)	—
Never	1697 (42.82)	401 (39.88)		416 (42.90)		423 (43.36)		457 (44.51)	—
Now	1158 (28.63)	277 (26.87)		285 (28.43)		296 (28.45)		300 (30.30)	—
Alcohol consumption, *n* (%)	—	—		—		—		—	0.01
No	475 (10.22)	148 (12.40)		130 (12.54)		107 (8.96)		90 (7.68)	—
Yes	3653 (89.78)	884 (87.60)		902 (87.46)		925 (91.04)		942 (92.32)	—
BMI (kg/m^2^), *n* (%)	—	—		—		—		—	<0.0001
<25	1187 (32.11)	163 (15.31)		197 (19.31)		280 (31.20)		547 (56.65)	—
25–30	1264 (29.05)	250 (22.92)		302 (29.26)		370 (32.54)		342 (30.43)	—
≥30	1677 (38.84)	619 (61.77)		533 (51.43)		382 (36.25)		143 (12.92)	—
Diabetes, *n* (%)	—	—		—		—		—	<0.0001
Borderline	92 (2.00)	27 (3.08)		30 (2.52)		23 (1.95)		12 (0.79)	—
No	3505 (89.19)	797 (80.26)		857 (88.62)		903 (91.82)		948 (94.12)	—
Yes	531 (8.81)	208 (16.66)		145 (8.86)		106 (6.23)		72 (5.10)	—
Cancer, *n* (%)	—	—		—		—		—	<0.0001
No	3617 (88.30)	866 (83.79)		908 (88.22)		906 (88.25)		937 (91.82)	—
Yes	511 (11.70)	166 (16.21)		124 (11.78)		126 (11.75)		95 (8.18)	—
Hypertension, *n* (%)	—	—		—		—		—	<0.0001
No	2413 (64.72)	487 (52.85)		553 (61.32)		619 (65.28)		754 (76.14)	—
Yes	1715 (35.28)	545 (47.15)		479 (38.68)		413 (34.72)		278 (23.86)	—
CVD, *n* (%)	—	—		—		—		—	<0.0001
No	3404 (86.36)	782 (78.83)		823 (84.29)		879 (90.18)		920 (90.47)	—
Yes	724 (13.64)	250 (21.17)		209 (15.71)		153 (9.82)		112 (9.53)	—
HEI‐2015,*n* (%)	—	—		—		—		—	0.03
Q1	1032 (25.92)	274 (28.80)		257 (26.59)		246 (25.43)		255 (23.58)	—
Q2	1032 (24.33)	277 (27.81)		266 (24.40)		254 (24.52)		235 (21.49)	—
Q3	1032 (25.32)	253 (23.72)		245 (25.08)		274 (26.32)		260 (25.85)	—
Q4	1032 (24.43)	228 (19.67)	264 (23.92)		258 (23.73)		282 (29.09)		—
EQ, *n* (%)	—	—	—		—		—		<0.0001
Q1	1034 (21.68)	317 (28.51)	270 (22.23)		254 (21.56)		193 (16.13)		—
Q2	1030 (24.43)	280 (29.05)	274 (25.20)		241 (23.02)		235 (21.50)		—
Q3	1032 (26.66)	244 (24.26)	257 (27.64)		253 (25.23)		278 (28.87)		—
Q4	1032 (27.24)	191 (18.18)	231 (24.94)		284 (30.18)		326 (33.49)		—

Abbreviations: BMI, body mass index; CALLY, C‐reactive protein–albumin–lymphocyte; CVD, cardiovascular disease; EQ, daily energy intake; HEI‐2015, Healthy Eating Index‐2015.

### 3.2. CALLY Index and All‐Cause Mortality in CIAD

Among the 4128 included CIAD patients, a total of 1068 all‐cause deaths occurred. We grouped the CALLY index into quartiles and used the lowest as the baseline to evaluate its association with all‐cause mortality in CIAD (Table 2). In Model 1, after adjusting for demographic variables such as gender, age, and race, we observed a significant negative dose–response relationship between the CALLY index and all‐cause death in CIAD patients (trend test, *p*  < 0.001); specifically, for every one‐quartile increase in the CALLY index, the risk of death decreased significantly. Model 2, further adjusted for sex, race, age, educational attainment, smoking status, PIR, insurance, alcohol consumption, daily energy intake, and HEI‐2015, revealed that compared with the lowest quartile, the highest CALLY quartile was linked to a 58% lower risk of all‐cause mortality (HR = 0.42, 95% CI: 0.32–0.53, *p* < 0.0001). Model 3, incorporating additional adjustments for BMI, hypertension, diabetes, cardiovascular disease, and cancer while maintaining all previous covariates, sustained this inverse association (HR = 0.36, 95% CI: 0.27–0.47, *p* < 0.0001). All analytical models confirmed statistically significant trend associations (all trend *p*‐values were less than 0.05), strongly indicating a consistent and robust negative correlation between higher CALLY levels and a reduced risk of all‐cause mortality in patients with CIAD.

**Table 2:** CALLY and mortality risk in adults with CIAD.

CALLY	Model 1	*p*	Model 2	*p*	Model 3	*p*‐Value
	HR (95% CI)		HR (95% CI)		HR (95% CI)	
All‐cause mortality						
Q1	Ref	Ref	Ref	Ref	Ref	Ref
Q2	0.60 (0.49–0.74)	<0.0001	0.63 (0.53–0.76)	<0.0001	0.64 (0.53–0.77)	<0.0001
Q3	0.49 (0.38–0.63)	<0.0001	0.53 (0.42–0.68)	<0.0001	0.54 (0.42–0.68)	<0.0001
Q4	0.36 (0.28–0.47)	<0.0001	0.42 (0.32–0.53)	<0.0001	0.36 (0.27–0.47)	<0.0001
*p* for trend	<0.0001	—	<0.0001	—	<0.0001	—
Cardiovascular mortality						
Q1	Ref	Ref	Ref	Ref	Ref	Ref
Q2	0.53 (0.37–0.76)	<0.001	0.53 (0.37–0.75)	<0.001	0.51 (0.37–0.71)	<0.0001
Q3	0.47 (0.34–0.67)	<0.0001	0.50 (0.35–0.73)	<0.001	0.57 (0.39–0.83)	0.003
Q4	0.34 (0.23–0.51)	<0.0001	0.39 (0.26–0.58)	<0.0001	0.33 (0.21–0.51)	<0.0001
*p* for trend	<0.0001	—	<0.0001	—	<0.0001	—

*Note:* Model 1 was adjusted for gender, age, and race only. Model 2 further incorporated educational attainment, smoking status, PIR, insurance, alcohol consumption, EQ, and HEI‐2015 in addition to the Model 1 covariates. Model 3 additionally included BMI, hypertension, diabetes, CVD, and cancer beyond the variables adjusted in Model 2.

Abbreviations: BMI, body mass index; CALLY, C‐reactive protein–albumin–lymphocyte; CIAD, chronic inflammatory airway diseases; CVD, cardiovascular disease; EQ, daily energy intake; HEI‐2015, healthy eating index‐2015; PIR, poverty–income ratio.

**Figure 2:**
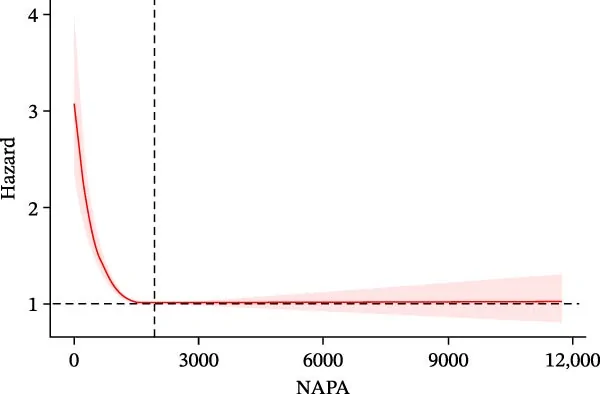
RCS showing the relationship between CALLY and all‐cause mortality.

Figure 2 illustrates the distribution of the CALLY index in the study cohort. After comprehensive adjustment for multiple covariates—including sex, race/ethnicity, age, educational attainment, smoking status, PIR, BMI, insurance, hypertension, alcohol consumption, diabetes, cardiovascular disease, cancer, daily energy intake, and HEI‐2015—RCS analysis revealed a significant linear association between CALLY levels and all‐cause mortality risk in CIAD patients (*p* < 0.05).

To further investigate the role of demographic characteristics in the CALLY index’s prediction of all‐cause mortality risk, we present the results of a detailed subgroup analysis for CIAD patients in Table 3. This analysis divided the population into subgroups based on gender, age, race, smoking status, BMI, and educational level with the aim of comprehensively assessing the consistency of the association between the CALLY index and all‐cause mortality across these subgroups. Compared with individuals in Q1 of the CALLY index, CIAD participants in the highest quartile (Q4) showed a significantly lower all‐cause mortality rate across all stratified subgroups (*p* < 0.05). However, interaction tests revealed that age (*p* < 0.0001) and sex (*p* = 0.03) had significant moderating effects on the association between the CALLY index and mortality risk. With the exception of age and sex, this association remained consistent across all other subgroups (including race, smoking status, BMI, and educational attainment) without any significant interactions being observed (interaction *p*‐values > 0.05).

**Table 3:** Subgroup analyses of CALLY and mortality risk in CIAD.

Characteristics	All‐cause mortality HR (95% CI):	*p*‐Value	*p* for interaction	Cardiovascular mortality HR (95% CI):	*p*‐Value	*p* for interaction
Gender	—	—	<0.0001	—	—	0.04
Male	0.14 (0.09–0.21)	<0.0001	—	0.13 (0.07–0.25)	<0.0001	—
Female	0.35 (0.25–0.49)	<0.0001	—	0.32 (0.19–0.53)	<0.0001	—
Age group	—	—	0.03	—	—	0.11
≤44	0.13 (0.05–0.34)	<0.0001	—	0.06 (0.01–0.52)	0.01	—
45–64	0.32 (0.21–0.49)	<0.0001	—	0.22 (0.09–0.56)	0.002	—
≥65	0.56 (0.42–0.73)	<0.0001	—	0.59 (0.39–0.89)	0.01	—
Race	—	—	0.15	—	—	0.11
White	0.24 (0.19–0.31)	<0.0001	—	0.24 (0.16–0.36)	<0.0001	—
Other	0.24 (0.15–0.39)	<0.0001	—	0.16 (0.08–0.34)	<0.0001	—
Smoking	—	—	0.38	—	—	0.63
Never	0.24 (0.15–0.38)	<0.0001	—	0.23 (0.11–0.48)	<0.0001	—
Former	0.31 (0.21–0.44)	<0.0001	—	0.28 (0.16–0.52)	<0.0001	—
Now	0.21 (0.13–0.33)	<0.0001	—	0.18 (0.07–0.49)	<0.001	—
BMI (kg/m^2^)	—	—	0.65	—	—	0.43
<25	0.22 (0.14–0.33)	<0.0001	—	0.23 (0.11–0.46)	<0.0001	—
25–30	0.17 (0.11–0.26)	<0.0001	—	0.16 (0.08–0.32)	<0.0001	—
≥30	0.29 (0.16–0.53)	<0.0001	—	0.33 (0.13–0.87)	0.02	—
Educational attainment	—	—	0.18	—	—	0.27
More than high school	0.20 (0.13–0.31)	<0.0001	—	0.16 (0.09–0.30)	<0.0001	—
High school diploma	0.24 (0.13–0.42)	<0.0001	—	0.47 (0.18–1.24)	0.13	—
Lower than high school	0.45 (0.32–0.65)	<0.0001	—	0.33 (0.19–0.60)	<0.001	—

*Note:* HRs compare Q4 with Q1 of the CALLY index; no covariates were adjusted.

Abbreviations: BMI, body mass index; CALLY, C‐reactive protein–albumin–lymphocyte; CIAD, chronic inflammatory airway diseases.

To mitigate potential survival‐time bias and competing mortality risks, this investigation excluded participants who died within 2 years postbaseline or experienced cancer‐related mortality. The results of the sensitivity analysis further confirmed the robustness of the above findings: after adjusting for relevant potential confounders, the negative association between the CALLY index and all‐cause mortality remained statistically significant (*p* < 0.05). As shown in Table 4, in the fully adjusted model, compared with participants in Q1, those in Q4 had a 66% significantly reduced risk of all‐cause mortality (HR = 0.34, 95% CI: 0.25–0.47, *p*  < 0.001), which fully confirms that higher CALLY levels provide a robust, independent protective effect for patients with CIAD.

**Table 4:** Sensitivity analyses.

CALLY	Model 1	*p*‐Value	Model 2	*p*‐Value	Model 3	*p*‐Value
	HR (95% CI)		HR (95% CI)		HR (95% CI)	
All‐cause mortality						
Q1	Ref.	Ref.	Ref.	Ref.	Ref.	Ref.
Q2	0.60 (0.46–0.77)	<0.0001	0.61 (0.49–0.76)	<0.0001	0.60 (0.48–0.75)	<0.0001
Q3	0.47 (0.35–0.63)	<0.0001	0.50 (0.37–0.67)	<0.0001	0.51 (0.38–0.69)	<0.0001
Q4	0.35 (0.26–0.47)	<0.0001	0.39 (0.29–0.53)	<0.0001	0.34 (0.25–0.47)	<0.0001
*p* for trend	<0.001	—	<0.001	—	<0.001	—
Cardiovascular mortality						
Q1	Ref.	Ref.	Ref.	Ref.	Ref.	Ref.
Q2	0.50 (0.33–0.78)	0.002	0.48 (0.31–0.75)	0.001	0.48 (0.31–0.73)	<0.001
Q3	0.45 (0.29–0.70)	<0.001	0.46 (0.29–0.71)	<0.001	0.53 (0.35–0.83)	0.005
Q4	0.30 (0.19–0.48)	<0.0001	0.32 (0.20–0.52)	<0.0001	0.31 (0.19–0.51)	<0.0001
*p* for trend	<0.0001	—	<0.0001	—	<0.0001	—

*Note:* Model 1 adjusted for sex, age, and race only. Model 2 further included education, smoking, PIR, insurance, alcohol, EQ, and HEI‑2015. Model 3 additionally incorporated BMI, hypertension, diabetes, CVD, and cancer based on the covariates in Model 2.

Abbreviations: BMI, body mass index; CALLY, C‐reactive protein–albumin–lymphocyte; CIAD, chronic inflammatory airway diseases; CVD, cardiovascular disease; EQ, daily energy intake; HEI‐2015, healthy eating index‐2015; PIR, poverty–income ratio.

### 3.3. CALLY Index and Cardiovascular Mortality in CIAD

**Figure 3:**
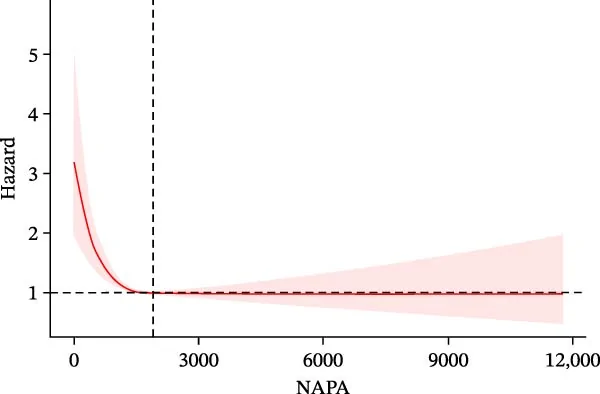
RCS analysis for CALLY and cardiovascular mortality.

This study analyzed cardiovascular mortality risk in 3442 patients with CIAD, among whom 362 cardiovascular deaths were recorded. Weighted Cox regression analysis revealed a significant inverse trend between increasing CALLY index quartiles and cardiovascular mortality risk when using the lowest quartile as a reference (*p* for trend < 0.001). After adjusting for multiple confounding factors, including sex, race, age, education level, smoking status, PIR, BMI, insurance status, hypertension, alcohol consumption, diabetes, cardiovascular disease, cancer, daily energy intake, and HEI‐2015, the fully adjusted model (Model 3) revealed that patients in Q4 exhibited a 67% reduction in cardiovascular mortality risk compared to the Q1 (HR = 0.33, 95% CI: 0.21–0.51, *p*  < 0.0001) (Table 2). RCS analysis further revealed that, after fully adjusting for the aforementioned confounding factors, the CALLY index was significantly and linearly inversely correlated with the risk of cardiovascular death (*p* < 0.05); see Figure 3 for details on this trend. Stratified analyses showed significant inverse associations in all subgroups except the high school‐educated subgroup (*p* = 0.12), while demonstrating significant effect modification by sex (interaction *p*‐values = 0.04); other subgroup analyses—including age, race, smoking, BMI, and educational attainment—yielded no significant interactions (all *p*  > 0.05) (Table 3). The validity of these results was confirmed by sensitivity analysis; the fully adjusted model revealed a 69% decrease in cardiovascular mortality risk for the highest versus lowest CALLY index quartile (HR = 0.31, 95% CI: 0.19–0.51, *p*  < 0.001) (Table 4). These findings clearly show that the CALLY index is a robust independent predictor of the risk of cardiovascular death in CIAD individuals, with particularly notable cardiovascular protective effects at higher index levels.

## 4. Discussion

Our findings indicate that higher CALLY index levels are significantly correlated with lower mortality rates in patients with CIAD. Following comprehensive adjustment for sociodemographic factors and baseline health conditions, analyses confirmed the CALLY index’s persistent predictor validity for both all‐cause and cardiovascular mortality in adult CIAD populations. Compared with conventional inflammatory markers like CRP, neutrophil count, and white blood cell count, this composite index integrates measures of systemic inflammation with nutritional status, showing enhanced predictive capability for assessing mortality risks. RCS analyses further validated these observations by revealing a characteristic L‐shaped dose–response relationship, indicating the index’s potential utility in stratifying high‐risk individuals susceptible to overall and cardiovascular‐specific mortality.

Chronic inflammation is closely associated with various chronic diseases, including cardiovascular disorders, diabetes, cancer, and respiratory conditions [19]. Persistent airway inflammation serves as the pathological foundation for CIADs, such as COPD and asthma [20, 21]. Tissue‐level chronic inflammation typically triggers immune cell recruitment from the circulation, thereby exacerbating inflammatory responses. Inflammatory markers, including leukocyte count, eosinophil count, and lymphocyte ratios, have demonstrated associations with asthma severity [22, 23]. Ke and colleagues [24] further confirmed that inflammatory biomarkers derived from complete blood counts in adults with asthma are significantly associated with an increased risk of all‐cause mortality and respiratory mortality [23]. Given the complex pathophysiology of CIADs, reliance on any single biomarker may prove insufficient for comprehensive health‐risk assessment. Paliogiannis et al. [25] systematically reviewed existing evidence, highlighting the clinical utility of the NLR in predicting COPD exacerbations. Furthermore, elevated systemic immune‐inflammation index (SII) levels have shown independent associations with both increased COPD incidence and all‐cause mortality [26]. Serum albumin, commonly employed to assess the nutritional status and visceral protein synthesis, demonstrates reduced production in systemic inflammatory states and malnutrition among critically ill patients. Previous investigations have revealed that decreased albumin and lymphocyte levels adversely affect prognosis across various malignancies, with lower serum lymphocyte and albumin concentrations predicting worse overall and relapse‐free survival. The LA index is actually the product of the lymphocyte count and albumin levels, and it has been shown to be useful for prognostic assessment [27], while the newly proposed CALLY index incorporates additional markers of chronic airway inflammation, thereby enabling a more comprehensive health‐risk assessment and showing promising potential as a prognostic indicator for CIADs.

This CALLY index represents an enhanced scoring system that integrates serum albumin levels (nutritional status), lymphocyte counts (immune function), and CRP levels (inflammatory response) to provide a comprehensive assessment of immune‐inflammation‐nutrition status. This cost‐effective, readily accessible, and easily calculable composite biomarker demonstrates significant clinical utility. CRP, an acute‐phase reactant, not only reflects systemic inflammation but also modulates innate immunity and inflammatory processes [28]. Meta‐analyses confirm that increased CRP levels are linked to severe exacerbations, hospitalization risk, and increased early mortality in CIAD patients [29]. Lymphocytes, as essential components of host defense mechanisms, serve as crucial indicators of immune competence [30], with lymphopenia and reduced lymphocyte counts representing established prognostic factors in COPD [31]. Serum albumin, a key nutritional marker in COPD, demonstrates significant clinical relevance as hypoalbuminemia independently predicts acute respiratory failure risk and increased mortality. These three biomarkers exhibit sensitivity to various physiological and pathological influences. Chronic airway inflammation is made worse by the complex interaction of immunology, nutrition, and inflammation. Our findings demonstrate that decreased CALLY index values (reflecting elevated CRP, reduced lymphocytes, and hypoalbuminemia) significantly associate with increased all‐cause and cardiovascular mortality risks in CIAD patients. This composite index provides superior prognostic value compared to individual biomarkers, offering a robust and reliable integrated assessment of disease severity that maintains strong predictive validity across multiple analytical models.

Our study has several advantages. First and foremost, we used a large, nationally representative sample to conduct an in‐depth exploration of the intrinsic relationship between the CALLY index and the risk of death among CIAD cases. This design significantly enhances the generalizability and applicability of our findings to the general U.S. population. In addition, we adjusted for a comprehensive set of covariates known to affect pulmonary function, which included age, smoking habits, physical activity level, BMI, and other confounding factors. The results demonstrated consistent stability even after adjusting for these potential confounders. However, it must be recognized that this study has certain limitations. First, because it employed an observational design, the data from this study can only reveal correlations and are not sufficient to establish a definitive causal relationship between the CALLY index and the risk of death from CIAD. Nevertheless, it’s important not to overstate the causal nature of our findings, given the inherent limitations of observational analyses and the possibility of residual confounding. On top of that, our dependence on self‐stated CIAD diagnoses may have introduced recall bias since participants’ recollections of their medical conditions could be incomplete or inaccurate. Additionally, the analysis did not incorporate certain CIAD risk factors, such as dyspnea severity and exacerbation history, due to the unavailability of relevant data. Due to limitations of the NHANES dataset, this study did not conduct disease‐specific analyses. In future studies, we will use real‐world clinical cohort data to examine the prognostic value of the CALLY index for asthma, COPD, and chronic bronchitis on a disease‐by‐disease basis.

## 5. Conclusions

Our findings demonstrate a significant association between elevated CALLY index levels and reduced risks of both all‐cause and cardiovascular mortality among CIAD patients. These observations suggest that the CALLY index may serve as a prognostic marker for mortality risk stratification in this population, though additional prospective and predictive performance analyses are needed to validate its clinical application.

**Nomenclature**
BMI: Body mass index
CALLY: C‐reactive protein–albumin–lymphocyte
CIADs: Chronic inflammatory airway diseases
CIs: Confidence intervals
COPD: Chronic obstructive pulmonary disease
CRP: C‐reactive protein
CVD: Cardiovascular disease
HRs: Hazard ratios
ICD‐10: International Classification of Diseases, 10th Revision
LA: Lymphocyte–albumin
NCHS ERB: National Center for Health Statistics Research Ethics Review Board
NDI: National Death Index
NHANES: National Health and Nutrition Examination Survey
NLR: Neutrophil‐to‐lymphocyte ratio
NPAR: Neutrophil‐to‐albumin ratio
RCS: Restricted cubic spline.

## Author Contributions

Yuting Fan, Baoqing Sun, and Zhiping Deng conceived the study and took primary responsibility for the final content. Yuting Fan and Zhao Chen performed the analyses and wrote the initial draft. Mingshan Xue, Wenqiang Li, and Youli Wen revised the manuscript.

## Funding

This work was supported by the Zigong Science and Technology Program (Grants 2023YKY07 and 2023YLWS21).

## Disclosure

All authors reviewed the final version and gave their approval for submission. The authors take full responsibility for the final content following a rigorous evaluation and editing process.

## Ethics Statement

This study has undergone ethical review and received formal approval from the Institutional Review Board of the U.S. Centers for Disease Control and Prevention (CDC) (Protocol Numbers: 2005‐06 and 2011‐17). All study procedures strictly adhered to local regulations and institutional policy requirements, and written informed consent was provided by the participants’ legal guardians or next‐of‐kin prior to enrollment.

## Consent

The authors have nothing to report.

## Conflicts of Interest

The authors declare no conflicts of interest.

## Data Availability

The data supporting the findings of this study were derived from the publicly available NHANES repository, which can be accessed at https://wwwn.cdc.gov/nchs/nhanes/. For additional information, please contact the corresponding author.

## References

[1] Chen Z. , Wen Y. , and Li W. , et al.Geriatric Nutritional Risk Index as a Predictor of Mortality in Women With Chronic Inflammatory Airway Disease: Evidence From NHANES. 1999-2018, Frontiers in Nutrition. (2025) 12, 10.3389/fnut.2025.1547952, 1547952. 40144569 PMC11939015

[2] GBD Chronic Respiratory Disease Collaborators , Prevalence and Attributable Health Burden of Chronic Respiratory Diseases, 1990-2017: A Systematic Analysis for the Global Burden of Disease Study 2017, The Lancet Respiratory Medicine. (2020) 8, no. 6, 585–596, 10.1016/S2213-2600(20)30105-3. 32526187 PMC7284317

[3] GBD 2015 Chronic Respiratory Disease Collaborators , Global, Regional, and National Deaths, Prevalence, Disability-Adjusted Life Years, and Years Lived With Disability for Chronic Obstructive Pulmonary Disease and Asthma, 1990-2015: A Systematic Analysis for the Global Burden of Disease Study 2015, The Lancet Respiratory Medicine. (2017) 5, no. 9, 691–706, 10.1016/S2213-2600(17)30293-X. 28822787 PMC5573769

[4] GBD 2019 Diseases and Injuries Collaborators , Global Burden of 369 Diseases and Injuries in 204 Countries and Territories, 1990-2019: A Systematic Analysis for the Global Burden of Disease Study 2019, Lancet. (2020) 396, no. 10258, 1204–1222, 10.1016/S0140-6736(20)30925-9. 33069326 PMC7567026

[5] Safiri S. , Carson-Chahhoud K. , and Noori M. , et al.Burden of Chronic Obstructive Pulmonary Disease and Its Attributable Risk Factors in 204 Countries and Territories, 1990-2019: Results From the Global Burden of Disease Study 2019, BMJ. (2022) 378, 10.1136/bmj-2021-069679, e069679. 35896191 PMC9326843

[6] Racanelli A. C. , Kikkers S. A. , Choi A. M. K. , and Cloonan S. M. , Autophagy and Inflammation in Chronic Respiratory Disease, Autophagy. (2018) 14, no. 2, 221–232, 10.1080/15548627.2017.1389823. 29130366 PMC5902194

[7] Barnes P. J. , Inflammatory Mechanisms in Patients With Chronic Obstructive Pulmonary Disease, Journal of Allergy and Clinical Immunology. (2016) 138, no. 1, 16–27, 10.1016/j.jaci.2016.05.011. 27373322

[8] Zhu N. , Lin S. , Wang L. , Kong X. , Huang W. , and Cao C. , Elevated Inflammatory Burden Index Increases Mortality in Adults With Chronic Inflammatory Airway Diseases: A Nationwide Cohort Study, BMC Pulmonary Medicine. (2024) 24, no. 1, 10.1186/s12890-024-03211-6, 399. 39164650 PMC11337749

[9] Thomsen M. , Ingebrigtsen T. S. , and Marott J. L. , et al.Inflammatory Biomarkers and Exacerbations in Chronic Obstructive Pulmonary Disease, JAMA. (2013) 309, no. 22, 2353–2361, 10.1001/jama.2013.5732. 23757083

[10] Lan C. C. , Su W. L. , Yang M. C. , Chen S. Y. , and Wu Y. K. , Predictive Role of Neutrophil-Percentage-to-Albumin, Neutrophil-to-Lymphocyte and Eosinophil-to-Lymphocyte Ratios for Mortality in Patients With COPD: Evidence From NHANES. 2011-2018, Respirology. (2023) 28, no. 12, 1136–1146, 10.1111/resp.14589. 37655985

[11] Di Vincenzo O. and Siotto M. , Editorial: Assessment of Nutritional Status in Chronic Diseases, Frontiers in Nutrition. (2025) 12, 10.3389/fnut.2025.1662137. PMC12380528 40880734

[12] Li Y. , Wei Q. , and Ke X. , et al.Higher CALLY Index Levels Indicate Lower Sarcopenia Risk Among Middle-Aged and Elderly Community Residents as Well as Hospitalized Patients, Scientific Reports. (2024) 14, no. 1, 10.1038/s41598-024-75164-z, 24591. 39426987 PMC11490578

[13] Xu Z. , Tang J. , Xin C. , Jin Y. , Zhang H. , and Liang R. , Associations of C-Reactive Protein-Albumin-Lymphocyte (CALLY) Index With Cardiorenal Syndrome: Insights From a Population-Based Study, Heliyon. (2024) 10, no. 17, 10.1016/j.heliyon.2024.e37197, e37197. 39296012 PMC11408039

[14] Zhu D. , Lin Y. D. , Yao Y. Z. , Qi X. J. , Qian K. , and Lin L. Z. , Negative Association of C-Reactive ProteIn-AlbumIn-Lymphocyte Index (CALLY Index) With All-Cause and Cause-Specific Mortality in Patients With Cancer: Results From NHANES. 1999-2018, BMC Cancer. (2024) 24, no. 1, 10.1186/s12885-024-13261-y, 1499. 39639229 PMC11619214

[15] Luo Z. , Chen S. , Chen P. , Qiu F. , Huang W. , and Cao C. , Oxidative Balance Score and Its Association With Chronic Inflammatory Airway Diseases and Mortality: A Population-Based Study, Frontiers in Nutrition. (2025) 12, 10.3389/fnut.2025.1541559, 1541559. 39911804 PMC11796618

[16] Brämer G. R. , International Statistical Classification of Diseases and Related Health Problems Tenth Revision, World Health Statistics Quarterly Rapport Trimestriel De Statistiques Sanitaires Mondiales. (1988) 41, no. 1, 32–36. 3376487

[17] Poverty Guidelines, Research, and Measurement, [https://aspe.hhs.gov/poverty-research, ].

[18] Cohrs S. , Rodenbeck A. , and Riemann D. , et al.Impaired Sleep Quality and Sleep Duration in Smokers-Results From the German Multicenter Study on Nicotine Dependence, Addiction Biology. (2014) 19, no. 3, 486–496, 10.1111/j.1369-1600.2012.00487.x. 22913370

[19] Furman D. , Campisi J. , and Verdin E. , et al.Chronic Inflammation in the Etiology of Disease Across the Life Span, Nature Medicine. (2019) 25, no. 12, 1822–1832, 10.1038/s41591-019-0675-0. PMC7147972 31806905

[20] Gopallawa I. , Dehinwal R. , Bhatia V. , Gujar V. , and Chirmule N. , A Four-Part Guide to Lung Immunology: Invasion, Inflammation, Immunity, and Intervention, Frontiers in Immunology. (2023) 14, 10.3389/fimmu.2023.1119564, 1119564. 37063828 PMC10102582

[21] Dong L. L. , Liu Z. Y. , and Chen K. J. , et al.The Persistent Inflammation in COPD: Is Autoimmunity the Core Mechanism?, European Respiratory Review. (2024) 33, no. 171, 10.1183/16000617.0137-2023, 230137. 38537947 PMC10966473

[22] Fahy J. V. , Type 2 Inflammation in Asthma—Present in Most, Absent in Many, Nature Reviews Immunology. (2015) 15, no. 1, 57–65, 10.1038/nri3786. PMC4390063 25534623

[23] Cazzola M. , Rogliani P. , Ora J. , Calzetta L. , and Matera M. G. , Asthma and Comorbidities: Recent Advances, Polish Archives of Internal Medicine. (2022) 132, no. 4, 10.20452/pamw.16250. 35485651

[24] Ke J. , Qiu F. , Fan W. , and Wei S. , Associations of Complete Blood Cell Count-Derived Inflammatory Biomarkers With Asthma and Mortality in Adults: A Population-Based Study, Frontiers in Immunology. (2023) 14, 10.3389/fimmu.2023.1205687, 1205687. 37575251 PMC10416440

[25] Paliogiannis P. , Fois A. G. , and Sotgia S. , et al.Neutrophil to Lymphocyte Ratio and Clinical Outcomes in COPD: Recent Evidence and Future Perspectives, European Respiratory Review. (2018) 27, no. 147, 10.1183/16000617.0113-2017, 170113. 29436405 PMC9488932

[26] Ye C. , Yuan L. , Wu K. , Shen B. , and Zhu C. , Association Between Systemic Immune-Inflammation Index and Chronic Obstructive Pulmonary Disease: A Population-Based Study, BMC Pulmonary Medicine. (2023) 23, no. 1, 10.1186/s12890-023-02583-5, 295. 37563621 PMC10416535

[27] Yamamoto T. , Kawada K. , and Hida K. , et al.Combination of Lymphocyte Count and Albumin Concentration as a New Prognostic Biomarker for Rectal Cancer, Scientific Reports. (2021) 11, no. 1, 10.1038/s41598-021-84475-4, 5027. 33658561 PMC7930240

[28] Yao Z. , Zhang Y. , and Wu H. , Regulation of C-Reactive Protein Conformation in Inflammation, Inflammation Research. (2019) 68, no. 10, 815–823, 10.1007/s00011-019-01269-1. 31312858

[29] Fermont J. M. , Masconi K. L. , and Jensen M. T. , et al.Biomarkers and Clinical Outcomes in COPD: A Systematic Review and Meta-Analysis, Thorax. (2019) 74, no. 5, 439–446, 10.1136/thoraxjnl-2018-211855. 30617161 PMC6484697

[30] Iseki Y. , Shibutani M. , and Maeda K. , et al.The Impact of the Preoperative Peripheral Lymphocyte Count and Lymphocyte Percentage in Patients With Colorectal Cancer, Surgery Today. (2017) 47, no. 6, 743–754, 10.1007/s00595-016-1433-2. 27783149

[31] Semenzato U. , Biondini D. , and Bazzan E. , et al.Low-Blood Lymphocyte Number and Lymphocyte Decline as Key Factors in COPD Outcomes: A Longitudinal Cohort Study, Respiration. (2021) 100, no. 7, 618–630, 10.1159/000515180. 33902057

